# What Determines the Sex of Children

**Published:** 1881-02

**Authors:** 


### WHAT DETERMINES THE SEX OF CHILDREN.

The first question is, When is sex determined? Is it at the time of conception or subsequently?

The answer to this appears to be given by the facts connected with double and triple births. It is in the human race a law, without exception, that twins embraced in the same chorion are of the same sex. Three explanations of this fact suggest themselves: 1. They are of the same sex because they are nourished by the same maternal blood; but this fails, because then twins must always be of the same sex, which they are not. 2. They are of the same sex because they are nourished from the same placental vessels; but this fails, because when one is amorphous, acephalous, or stunted the sex remains the same. 3.

That the sex was determined at the moment of conception; which is the only theory left. But what is true of twins, must be true of single births! therefore, we reach the decision that sex is fixed by the circumstances of conception. Such is the reasoning of Dr. Mayerhofer (*Von der Zeugung des Menschen*), and it seems conclusive.

The next question is, are the ovula or spermatozoa divided sexually, so that a given ovum or spermatozoon can only produce one sex, or is it a matter which depends on the relative condition of the parents at the time of cohabitation?

The former theories have been often maintained. Prof. Schultze teaches that the ovula in the ovary are fitted, some to develop only male, others only female infants. The ancients thought that the secretion of the one testicle produced males, the other females.

Both these and all similar theories cannot stand criticism. A very extensive and careful study of marriages, occurring over Germany, France and England, conducted by independent statisticians, leads directly to the formulating of the following rule: *Whichever party to coition is at the time in the fullest possession of his or her reproductive powers will determine in the embryo his or her sex.*

The facts are, that quite young men marrying women older than themselves, and quite old men marrying vigorous women, both beget more girls than boys. Husbands, as a rule, being older and more vigorous than their wives, as a rule more boys than girls are born (106 boys; 100 girls). When those marriages are taken in which the husbands are all older, but not more than ten years older than their wives, the population of children is about 700 boys to 600 girls.

There is hardly any doubt that the physical condition of the parent at the time of conception influences the sex in the direction that the most vigorous sex tends to perpetuate itself. It is part of this rule that when the male cohabits rarely, and thus has more vigorous and numerous spermatoza, the children are apt to be male.—*Phil. M. and S. Reporter.*

